# Advanced accident research system based on a medical and engineering data in the metropolitan area of Florence

**DOI:** 10.1186/1471-227X-13-3

**Published:** 2013-03-14

**Authors:** Simone Piantini, David Grassi, Marco Mangini, Marco Pierini, Giovanni Zagli, Rosario Spina, Adriano Peris

**Affiliations:** 1 Department of Mechanics and Industrial Technology, University of Florence, Florence, Italy; 2 Post-graduated School of Anesthesia and Intensive Care Unit of Emergency Department, Careggi Teaching Hospital, Florence, Italy; 3 Anesthesia and Intensive Care Unit of Emergency Department, Careggi Teaching Hospital, Florence, Italy

**Keywords:** In-depth accident database, Injury mechanism, Injury pattern, Biomechanics, Road accident, Injury causes

## Abstract

**Background:**

In the metropolitan area of Florence, 62% of major traumas involve powered two wheeler rider and pillion passengers, 10% cyclists, and 7% pedestrians. The urban and extra-urban areas are the most dangerous for the vulnerable road user. In-depth investigations are needed for assessing detailed information on road accidents. This type of study has been very limited in time frame in Italy, and completely absent in the Tuscan region.

Consequently a study called “In-depth Study of road Accident in FlorencE” (In-SAFE) has been initiated.

**Methods:**

A network between the Department of Mechanics and Industrial Technologies (University of Florence) and the Intensive Care Unit of the Emergency Department (Careggi Teaching Hospital, Florence) was created with the aim of collecting information about the road accidents. The data collected includes: on-scene data, data coming from examination of the vehicles, kinematics and dynamic crash data, injuries, treatment, and injury mechanisms. Each injury is codified thorough the AIS score, localized by a three-dimensional human body model based on computer tomography slices, and the main scores are calculated. We then associate each injury with its cause and crash technical parameters. Finally, all the information is collected in the In-SAFE database.

**Results:**

Patient mean age at the time of the accident was 34.6 years, and 80% were males. The ISS mean is 24.2 (SD 8.7) and the NISS mean is 33.6 (SD 10.5). The main road accident configurations are the “car-to-PTW” (25%) and “pedestrian run over” (17,9%). For the former, the main collision configuration is “head-on crash” (57%). Cyclists and PTW riders-and-pillions-passengers suffer serious injuries (AIS3+) mainly to the head and the thorax. The head (56.4%) and the lower extremities (12.7%) are the most frequently injured pedestrian body regions.

**Conclusions:**

The aim of the project is to create an in-depth road accident study with special focus on the correlation between technical parameters and injuries. An in-depth investigation team was setup and is currently active in the metropolitan area of Florence.

Twenty-eight serious road accidents involving twenty-nine ICU patients are studied. PTW users, cyclist and pedestrians are the most frequently involved in metropolitan accidents.

## Introduction

Despite the fact that during the period 2000–2010 road fatalities in Europe (EU27) have been reduced by 42.8% [1], in 2010 about 31.000 people were killed in road accidents, and about 300.000 were seriously injured. During the same period of time, Italy reduced the total number of victims by the 42.4%, but the number of injured people (light and serious) is still very high (about 300.000 in 2010) [2].

Vulnerable Road Users (VRU) (pedestrians, cyclists and PTW rider and pillion passenger) today are still at a very high risk of sustaining serious injuries, or being in a fatal accident, especially in metropolitan areas. Medical information on people admitted in a Tuscan Region Intensive Care Unit, and not dead on-scene of accident, is stored in the TTR [3-5] database. The 2009 and 2010 data of the TTR shows that 65% of severe injuries in the region are caused by road accidents. Twenty-nine percent of severe injuries occurred in non-urban areas and the majority (33%) in urban areas. In the metropolitan area of Florence, 62% of the severe injuries involved PTW rider and pillion passengers, 20% car occupants, 10% cyclists, and 7% pedestrians. The most frequent serious accident configurations are vehicle to vehicle accidents (73%), and run over pedestrians (18%).

Other important features are the permanent consequences sustained by people subjected to serious injury. A six-month follow-up after the traumatic event highlights that 7% of the people die, 2% remain in a vegetative state, 18% and 32% suffered, respectively, a serious and moderate disability, while 41% show a good recovery.

The analysis of the state-of-the-art shows that in-depth knowledge of real road accident data is very important for the comprehension of accident causation, mechanism of injury, and injury patterns [6]. Today the effects of accidents on car occupants and vulnerable road users are much better known than in the past, thanks to crash tests and computer simulation techniques.

The aim of crash tests is to provide qualitative or quantitative data, the first regarding the body parts that have impacted with some area of the passenger compartment or external vehicle and the kinematic followed by the occupants, while the second regards the acceleration and force parameters on each body region of the test dummies.

All this information is useful to understand the body part injured and the computation of the injury criterions, i.e. the Head Injury Criterion (HIC) and the Neck Injury Criterion (NIC) for the head and neck regions [7,8].

From these it is possible to calculate the probability of having a lesion corresponding to a given AIS score respective to these body regions.

In the crash tests, Anthropometric Dummies (AD) or Post Mortem Human Subject (PMHS) are used for the evaluation of the injuries, while in the computer simulation techniques Multi-Body Human Models (MBHM) or Finite-Element Human Models (FEHM) are used respectively. However, the AD and MBHM are not completely satisfactory give that the capability of the dummies to reproduce human behaviour, and particularly the injury description, is limited [9]. But also for the PMHS, the correlation with real injuries does not always correspond to the real outcome, due to the condition of the PMHS, e.g. inactive muscles, decomposition, positioning and support, age, height, weight.

In-depth accident studies allow the monitoring of the injuries sustained by the people involved in serious road accidents, in term of type, localization, frequency and severity compared to vehicle and crash configurations, objects impacted, and so on.

This type of research gives the possibility to relate real accident situations, as well as crash tests. Structures causing injuries can be recognized at an early stage. Feedback regarding the road traffic engineering can also be obtained.

The data is also used for recognizing and assessing potential areas of future safety developments, evaluating vehicle safety performance in real world accident situations, and supporting and validating computer simulations. For example, statistical data on important factors, e.g. impact speed, angle, and mass, can be used as the basis for defining standards for impact tests.

Some of the main real world in-depth accidents studies across Europe include the “German In Depth investigation Accident Study” (GIDAS) [10] in Germany, the Co-operative Crash Injury Study (CCIS) [11] and “On The Spot” (OTS) [12] in the United Kingdom, the “In-Depth Car Accident Analysis” (EDA) of INRETS in France [13] and the SafetyNet project operating until 2008 in six European countries [14]. For the in-depth study of road accidents focused on the PTW, the “Motorcycle Accident In depth Study” (MAIDS) [15] project is the reference for this type of vehicles.

In the United States the “National Accident Sampling System “(NASS) [16] and the “Crash Injury Research and Engineering Network“ (CIREN) [17] are the main in-depth accident research systems, and in Japan there is a collaborative study by “Japan Automobile Research Institute“ (JARI), Nippon Medical School Chiba Hokuso Hospital, and the “Institute for Traffic Accident Research and Data Analysis” (ITARDA) [18].

All this information can be useful for a wide range of fields of research such as ‘vehicle design for active and passive safety,’ ‘biomechanics,’ ‘driver behaviour,’ ‘trauma medicine,’ ‘road design,’ and so on. The data is also used for recognizing and assessing potential areas of future safety developments, evaluating vehicle safety performance in real world accident situations, and supporting and validating computer simulations. For example, statistical data on important factors, e.g. impact speed, angle, and mass, can be used as the basis for defining standards for impact tests, but also to develop new devices or shapes to mitigate the injuries, to improve current triage operations, to develop and validate new tools for the prediction of the severity of the injuries [19,20] and to evaluate the change produced by the countermeasures adopted.

In Italy, the collection and study of in-depth real world accident data has been very time limited in the past, and completely absent in the Tuscany region. The projects conducted in Italy are the MAIDS project, led in the Pavia province between 1999 and 2001 and focusing on PTW vehicles, and the SafetyNet project conducted in the Marche region between 2004 and 2008, where all types of road accident data were collected.

Due to the importance of the data coming from this type of study and the current absence in Italy of similar research, a medical-engineering network has been created.

In the “methods” section, our modus operandi is explained, and a case study is introduced step-by-step. In the "results" section, the main results on the analysis of the road accidents currently studied are shown. In the "discussion" section, some preliminary consideration deductible from the previous results are highlighted.

## Methods

The study is based on the direct collaboration between the Department of Mechanics and Industrial Technologies at the University of Florence (Italy) and the ICU of the Emergency Department (Careggi Teaching Hospital, Florence, Italy), and, indirectly, with police forces involved in the road accident detection, the Emergency Medical System (EMS) of Florence and the Emergency Room (ER) of the Careggi Teaching Hospital [21].

Internal Review Board waived the need of ethical approval due to the nature of the study. And the the aim of this research is to conduct an in-depth investigation into road accidents that have generated severe injuries (major trauma and potential ones) in the metropolitan area of Florence, and to reconstruct the causes and the mechanisms of the injuries. Moreover, the study aims to collect information regarding the disabilities sustained by the injured in order to evaluate their social costs, and also to determine what changes and improvements to vehicle design might mitigate or prevent these injuries in the future. To this purpose, a network of physicians, statisticians and engineers was established to link environmental data acquired on the scene of the accident with crash parameters and clinical information about the injuries.

The study selected all the road accidents where at least one of the persons involved was admitted to the ICU with a diagnosis of major trauma, i.e. an ISS greater than 15. None dead on-scene or in the ER case were collected in this study. The working team, named In-SAFE team, is composed by ICU physicians, engineers and statisticians.

### Sampling area and representatives

**Figure 1:**
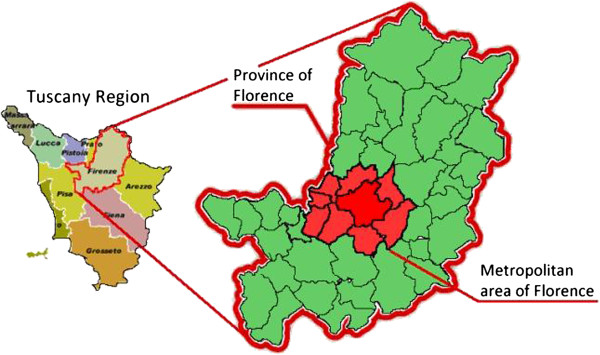
Sampling area.

The road accidents analyzed in this study were in the metropolitan area of the city of Florence. This area is made up of nine municipalities, covers a surface of 466 km2, with a population of approximately 604.000 people (Figure 1). The sampling area is mainly composed by urban zones and in small part by extra urban areas.

Since 2005, the trauma network of the Tuscan Region has organized the ICU, which works on major trauma through the hub/spoke system. For the Province of Florence, the hub hospital of reference is the Careggi Teaching Hospital which receives all major traumas of patients that are more than 16 years old.

**Figure 2:**
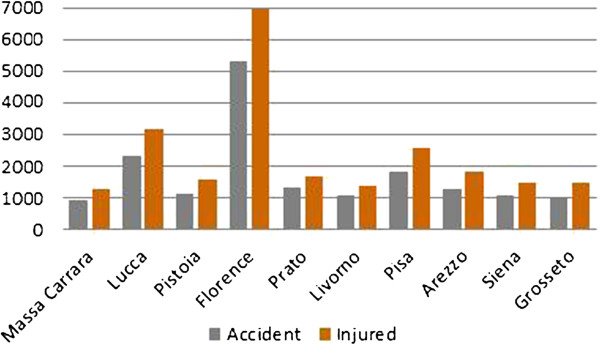
Number of road accidents and injured in Tuscany for 2010.

**Figure 3:**
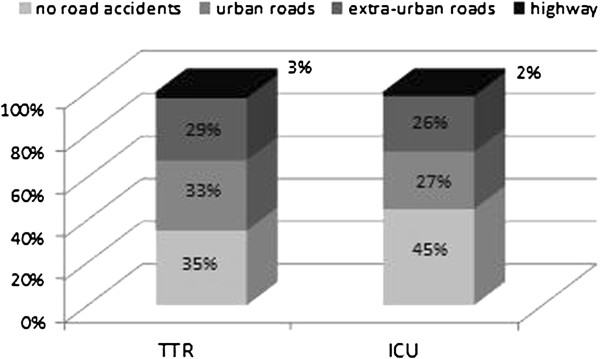
Number of major trauma in Tuscany and at the Careggi University Hospital for 2010.

In 2010, Florence was the province with highest number of road accidents and injured in Tuscany (Figure 2). Sixty-five percent of the major traumas in Tuscany were caused by road accidents, and only 3% of these occurred on highways. The access for major trauma to the ER of the Careggi Teaching Hospital confirms the regional trend (Figure 3). Therefore, the metropolitan area selected should ensure that the distribution of the sample is similar to the TTR.

### An in-depth multidisciplinary investigation

**Figure 4:**
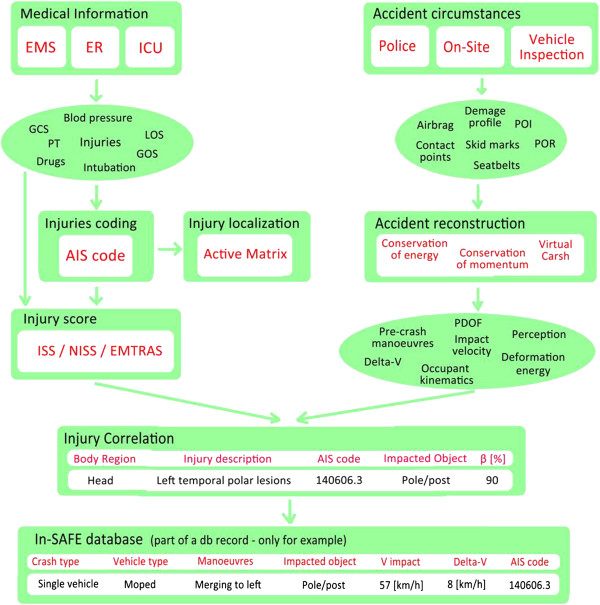
Flow chart of phases and data of the study.

With the cooperation of the police forces, the In-SAFE team acquires general information about: the *crash scene*, e.g. point of impact, point of rest; *description of the environment*, e.g. roadway configuration, traffic control data, weather conditions; *the vehicle*, e.g. type and model, engine size; and *people involved in the crash*, e.g. gender, age, type of licence and so on. In the following the main phases of the study are outlined. They are also shown in Figure 4.

### On-site investigation

The team collects more detailed information, such as skid marks, debris, deposit of liquids, point of rest of the vehicle, line of sight of each vehicle’s driver/rider or people involved in a crash, in order to substantiate the exact point of impact.

### Vehicles examination

**Figure 5:**
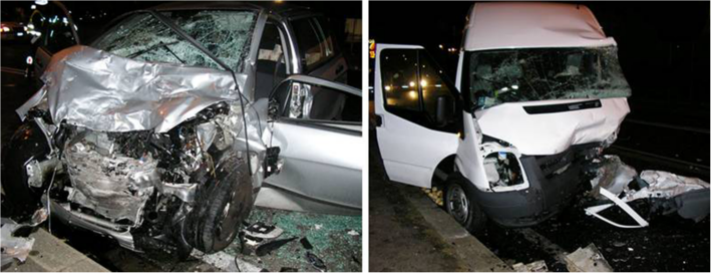
On scene and vehicle damage photography documentation.

Each vehicle involved in the accident is carefully examined by the In-SAFE team. All damage (direct or indirect) or contact points are photographed (Figure 5).

#### Exterior parts

The damage profile is quantified measuring the damage width. The latter is subdivided in six parts (C1-C6), where the dimension of the damage is quantified (CRASH3 method) [22,23]. In order to describe the nature and the location of the direct contact on the vehicle in car and van accidents the Collision Deformation Classification (CDC) [24] is used. For accidents involving medium and heavy trucks, and articulated combinations, the Track Deformation Classification (TDC) [25] is used.

The Wraps Around Distance (WAD) measurement for determination of the pedestrian or cyclist interaction with the vehicle is also acquired. Finally, for the PTW, the wheelbase shortening is collected.

#### Interior parts

**Figure 6:**
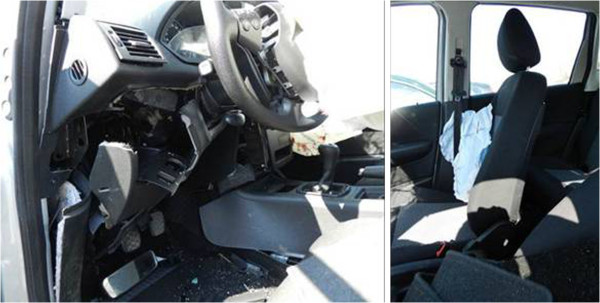
Interior vehicle photography documentation.

Vehicle interiors are thoroughly investigated for evidence of occupant contacts, and to quantify the intrusions. These data are then stored using the Passenger Compartment Classification (PCC) developed by Standardization of Accident and Injury Registration Systems (STAIRS) project [26]. Special attention is given to the usage of the seatbelt, activation of the pretensioner, and airbag activation (Figure 6).

### Accident reconstruction methodologies

From the previously collected data, the accident is reconstructed to evaluate the accident dynamics and the main physical parameters concerning the crash phase, as well as pre-crash phase manoeuvres, such as avoidance actions.

The post-crash velocity of each vehicle involved in a crash is evaluated by means of the analysis of the post-crash motion. The deformation energy and the velocity variation (ΔV) are estimated through Crash3 method [27]. The impact velocity of each vehicle is assessed by applying the principles of energy balance and the momentum analysis. By the use of crash simulation software (PC-Crash 8.3 and Virtual CRASH 2.2) all the previous data are verified and validated, and other parameters, such as the PDOF and the impact angle between the vehicles, are also evaluated.

In order to assess the range of uncertainty of the analysis, the Finite Difference Method (FDM) [28] is used. This is a numeric approach to partial differentiation of the equation used. The method consists in the calculation of the uncertainty range around the nominal value.

### Injury and physiological derangement evaluation

The medical data collected in the database are selected to provide a clear correlation between the trauma’s dynamic and the injury’s localization and severity.

The main information coming from the EMS (e.g. Glasgow Coma Scale, blood pressure, and intubation) and ER/ICU (e.g. diagnostics), the AIS and ISS scores, the EMTRAS and the Computed Tomography information, are the scores and data chosen for the previous aims.

The AIS was developed by the Automotive Committee On Medical Aspects of Automotive Safety in 1971 [29]. The last revision of the score is the AIS 2005, updated in 2008. Because the different AIS versions are not always compatible, injury severity scoring tools using the new AIS should be compared to those using previous versions in terms of score and predictive performance [30]: Carroll et al. show a reduction in traumatic brain injury (TBI) AIS when recorded using the 2005 revision versus the 1998 one [31]. For this reason, the In-SAFE database includes the AIS 2005 and AIS 1998 codifications, in order to asses differences in trauma severity classifications, and to allow the comparison with other databases using both revisions of the AIS. The ISS was introduced by Baker in 1974 to classify the severity of traumas involving lesions in more than one of the AIS regions. The score is calculated summing the square of the three highest AIS of three different body regions. No more than one AIS can be taken from a single region [30,31]. [30,31]. If a lesion is graded as 6, the ISS is automatically calculated as 75. This choice put greater attention on the multiplicity of trauma injury but at the same moment it can overlook multiple lesions suffered by the same part of the body. For this reason in 1997 Osler et al. developed the NISS, which is calculated summing the square of the 3 highest AIS, without any regard to the body region [32,33]. The authors affirm the superiority of the NISS to the ISS to predict the outcome of the trauma patient, and this conclusion is supported by Lavoie et al. [34]. In addition, for research purposes, the EMTRAS score, a new trauma score developed in Germany in 2009 that is calculated by using the age of the patient, the on-scene GCS, the Base excess, and the Prothrombin Time at the ER [35], has been added to the In-SAFE database. Drug and alcohol abuse are a major cause of loss of life, threatening injury in motor vehicle accidents, both in the US and in Europe [36,37]. Drugs test includes ethanol, cannabis, cocaine, amphetamine, benzodiazepine, barbiturate, and opioids dosage, collected upon admission in the ER, and recorded in In-SAFE. Ethanol was measured with head-space gas chromatography/mass spectrometry, whereas cannabis, cocaine, amphetamine, benzodiazepine, barbiturate ,opioids were dosed using Enzyme-Linked ImmunoSorbent Assay). To avoid false positives, daily internal control dosage are performed, and in case of a patient with elevated concentration of a substance (in absence of a known addiction), the analysis is repeated Moreover, on scene drugs are recorded, as well as first aid medical treatments.

The impact of road accident dynamics and lesions on the outcome are studied by recording length of stay, mortality at 6 months, and the follow-up program at 6 months on the ICU database. As an indicator of the quality of life recovered at 6 months after the event (follow-up at 6 months) the Glasgow Outcome Scale (GOS) [38] is used, as well as the questionnaire EuroQol5 EQ5-D with scale EQ5-D-VAS [39], which includes a medical examination. In case a patient cannot sustain a medical visit, a telephone interview is performed. Patient pre-accident drug treatment and pre-existing medical conditions seem to correlate with worse outcome, in terms of complication, ICU and Hospital length of stay, and lower functional outcome [40-43]. For this reason these data are recorded in a dedicated section of the database that includes the type and number of pre-existing medical conditions, and the type and dosage of each drug (ethanol, cannabis, cocaine, amphetamine, benzodiazepine, barbiturate, opioids). Despite some limitations due to risk related to ionizing radiation, CT remains the most sensitive imaging exam to assess trauma lesions [44-47]: for this reason for head, neck, face, chest and abdomen CT slices are chosen.

**Figure 7:**
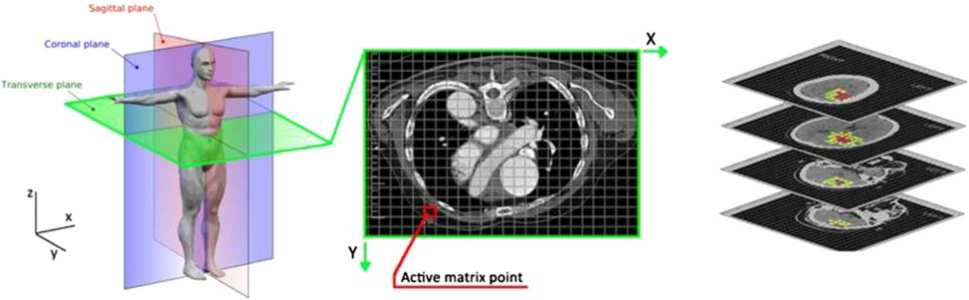
Graphical method for the active injuries’ localization.

In addition to the encoding of each lesion using the AIS code, these are identified by means of a three-dimensional localization tool that uses a discretization of the human body based on a set of CT slices equipped with an active matrix (Figure 7).

This was done by dividing a human body not affected by clinical pathologies through cross sections of CT scan made at regular intervals in the sagittal plane (z axis). Each slice (or plane) is divided into a point’s matrix. In this way, each point has its Cartesian coordinate (x, y, z) fixed, where x and y are read in the transverse plane (CT slice) while the z coordinate is the height of the CT slice, with zero value at top of the head. The matrix dimension depends on of the size of the section. The body regions head-face, neck, thorax, and abdomen are divided, respectively, into 8, 3, 15 and 13 slices. For the facial bones, vertebrae, rib cage, pelvis, and limbs, an active matrix built on the anatomical atlas figure is used to localize lesions with more sensitivity.

This type of localization of the lesions, for example, provides a means to compare the distribution of the damage (in terms of extent of the lesion) among different people, or even to realize the frequency distributions of the damage (mean and standard deviation) relative to a certain region of the body. More generally, it provides the possibility of correlating the area of damage with other types of information (i.e. impact velocity or direction, type of crash).

### Injury correlation phase

This phase is the heart of the study but also the most complex and subjected to errors. In this stage, the kinematics and dynamics of vehicles and people involved and the injuries are correlated. The injury information is assessed mainly by CT scan performed at the admission in the ER; other imaging exams (i.e. vascular CT Scan, Magnetic Resonance Imaging) can be added to CT to identify specific lesions.

The dynamic and kinematic information of the vehicles and people involved are assessed through physical principles and software. Once the injuries and dynamics are clearly identified, a meeting between intensive care physicians and engineers is organized in order to correlate each injury to its cause. By merging the data previously gathered and using state-of-the-art biomechanics of impact, it is possible to understand cause and mechanism of injuries.

In the end, for each association, the definition of a level of reliability of the correlation process (β), in percentage, indicates the quality of the data produced.

The reliability is defined as

β=1−a

where α is the uncertainty that we have about the association (injury vs. cause).

During the data analysis phase, a threshold value, fixed in β = 60%, is used for the selection of the most significant associations (Table 1).

**Table 1:** Summary of the correlation results between injuries and causes

**Body region**	**Injury description**	**AIS code**	**Impacted object**	**β [%]**
Head	Left temporal polar lesions	140606.3	Pole/post	90
Head	Millimetric left frontal parietal subdural hemorrhage	140651.3	Pole/post	90
Head	Widespread cerebral oedema	140670.3	Asphalt	90
Head	Right temporal parietal occipital depressed fracture	150404.3	Asphalt	90
Head	Right temporal styloid process fracture	150402.2	Asphalt	90
Head	Right tympanic and petrous fracture with hemotympanum	150202.3	Asphalt	90
Head	Right temporal-parietal-occipital multiple fractures depressed in the occipital region and diastatic in the mastoid region	150202.3	Asphalt	90
Head	Lacerated and contused right temporal parietal (2,5 cm) lesions	140616.4	Asphalt	90
Head	Pneumocephalus bubbles	140682.3	Asphalt	90
Head	Peri mesencephalic subarachnoid haemorrhage, with relative encephalic pons and mesencephalic hypodensity	140695.3	Asphalt	70
External	Contused and lacerated wounds to the face, hematoma lateral	910400.1	Asphalt	40
Thorax	Contusion of the right upper lobe. Right paravertebral inferior lobe and left paravertebral inferior lobe contusion.	441412.4	Asphalt	90

### Data stored system

**Figure 8:**
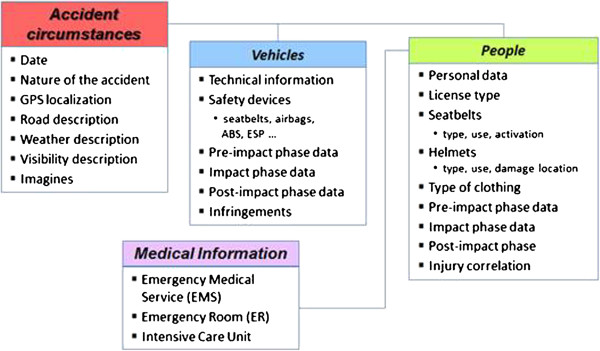
Database In-SAFE – Main clustering of data collected.

All the data collected are stored in a relational database (In-SAFE), where the variables are coded in accordance with the state-of-the-art techniques. The standardized protocols taken as reference are the Common International Methodology for in-depth accident investigation (OECD) [48,49] and STAIRS project [26]. The In-SAFE database contains about 700 variables divided in three main groups: environment, vehicles and people. The people group contains both demographic and medical information (Figure 8).

### Correlation analysis between injuries and dynamics: a case study

**Figure 9:**
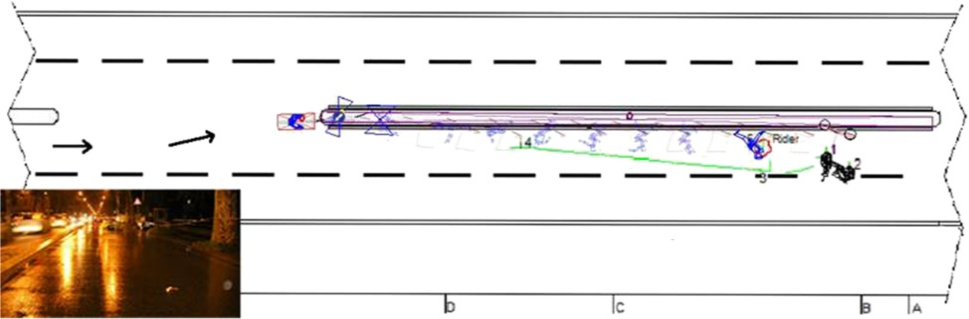
Scene of the accident, with point of impact and point of rest of rider and moped.

This accident, which occurred on an urban road, involved a 26 year old rider of a moped (scooter style) in a head-on collision against a road sign (single vehicle accident). Informed consent to publish this case and any accompanying images was obtained from the next of kin of the patient. The road was straight and divided into two roadways separated with a curb indicated by the road sign, as seen in Figure 9.

The rider, with a positive blood alcohol level (2.6 g/l), was riding at night (with road illumination) and heavy rain conditions. The moped was equipped with a windshield. Due to the high blood alcohol level (in this case the primary cause of the accident), the rider failed to keep a straight trajectory and collided with the road sign (1st impact). After a flying phase both the moped and rider impacted with the ground (2nd impact) and continue with a sliding phase before stopping. The total distance covered by the scooter from the point of impact to the point of rest was about 25 m, while the total distance covered by the rider was about 21 m.

Applying the equation of the launched ballistic proposed by Searle [48] it is possible to estimate the impact velocity of the moped (62 ± 5 km/h) and through computer simulation it is possible to reconstruct a 3D scenario of the accident and refine and validate the crash parameters, such as the impact velocity (57 ± 5 km/h) and the delta-V (8 ± 3 km/h).

The moped used for the computer simulation is a generic scooter modelled as a rigid body, resized in terms of mass, wheelbase, and dimension of the wheels. The rider is modelled as a multibody human model available in the software. Comparing the POR of moped and rider obtained with the software and those measured (points 1, 2, 5), as seen in Figure 9, it is possible to see the good quality of the computer simulation performed with the software. The rest position of the rider reconstructed with the software is in good agreement with the actual final position, while the moped one is relatively good but does not perfectly match with the actual position, probably due to the simplified model used to represent the moped and mainly in the modelling of the first impact.

The rider was wearing a demi-jet helmet that became detached after the first impact. For this reason, during the impact against the ground, he sustained serious head injuries and eventually died 47 days after the accident.

The Maximum AIS (MAIS = 4) sustained by the rider is in the head/neck body region and thorax body region, and the ISS score is equal to 33 (Table 2).

**Table 2:** Summary of the injury severity score for the rider

	**MAIS**
Head or neck	4
Face	0
Thorax	4
Abdomen	0
Extremities	0
External	1
ISS	33

**Figure 10:**
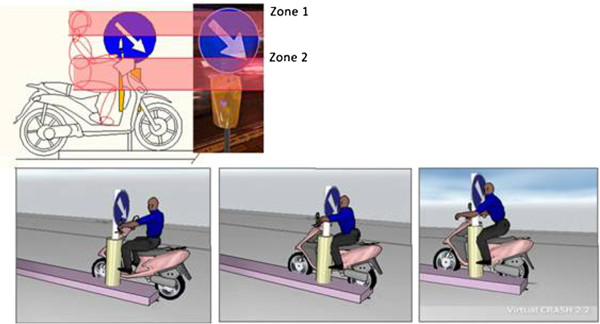
Impact against road sign (1st impact).

**Figure 11:**
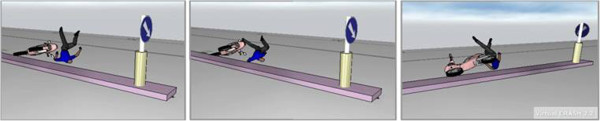
Rider impact on the ground (2nd impact).

In agreement with the on-scene and vehicle investigation and reconstruction, in the first impact the rider crashes with the front-left side of the moped and with his head striking against the yellow part (zone 1) and the blue part of the road sign (zone 2) (Figure 10). After this impact, rider and moped begin a flying phase which ends with landing on the ground and the subsequent slide to the rest position. In this phase, the rider impacts his head and then his thorax on the ground (Figure 11).

**Figure 12:**
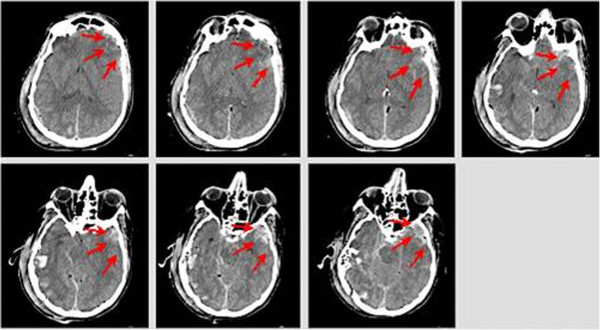
Head injuries – impact against road sign.

As a consequence to the first impact (against the road sign) with the helmet on, the rider sustained the following injuries: left temporal polar lesions (2.5 cm) with millimetric left frontal parietal subdural hemorrhage (Figure 12).

The subdural hematoma (or hemorrhage) is classified as a focal TBI i.e. a coup effect. This is caused by the compressive stresses that are generated when there is a relative motion of the brain with respect to the inner surface of the cranial cavity due to the inertial effects. As a consequence of the detached helmet, the impact against the ground occurs without any protection, causing the most serious head injuries. Ground contact also accounts for the thoracic injuries.

**Figure 13:**
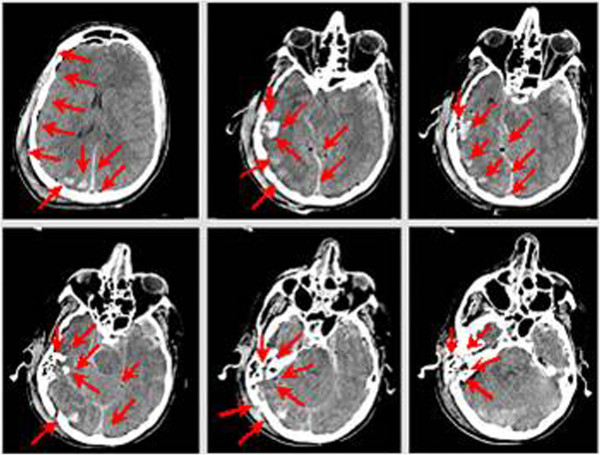
Head injuries – impact against the ground.

The main head injuries highlighted by CT scan (Figure 13) are: right temporal-parietal-occipital multiple fractures, depressed in the occipital region and diastatic in the mastoid region; diastatic skull base clivus fracture, involving sphenoid bone body and both carotid channel; right temporal styloid process and right tympanic fracture; right petrous fracture with hemotympanum; pneumocephalus bubbles; lacerated and contused right temporal parietal (2.5 cm) lesions; peri mesencephalic subarachnoid haemorrhage, with relative encephalic pons and mesencephalic hypodensity and widespread cerebral oedema.

The depressed skull fractures are caused by the direct contact with the ground that has generated a high deformation of the skull. This is due to the minor lateral strength of the skull with respect to its frontal and rear regions [50,51].

**Figure 14:**
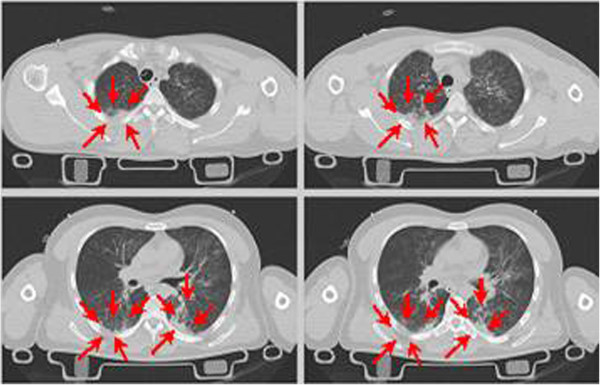
Thorax injuries – impact against the ground.

A right upper lobe lung contusion and bilateral lower lobe lung contusion in the paravertebral area are also sustained in the thoracic region (Figure 14). Both injuries are caused by the compression of the lung at high impact velocity.

A summary table with all correlation results and level of reliability in percentage values is shown in Table 1.

## Results

Twenty-eight serious road accidents occurred between January through July 2011 in the metropolitan area of Florence are included in this study.

### Demographics of injured

**Figure 15:**
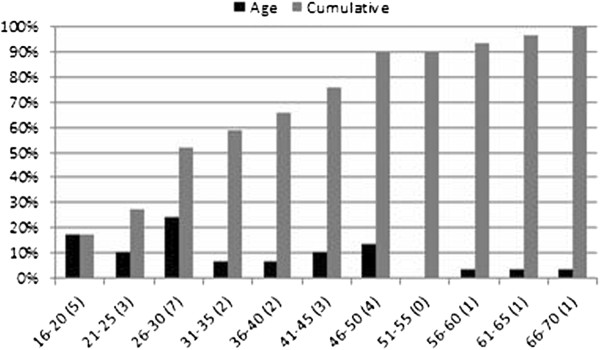
Age distribution of major trauma in In-SAFE database.

The mean age at the time of accident was 34.6 (SD 13.9) (range 16–70 years) and the people most affected are between 26 years and 30 years. About 70% of severely injured people are younger than 45 years (Figure 15). Male subjects constituted 83% (n = 24) and female subjects 17% (n = 5).

PTW riders-and-pillions-passengers are 41% (n = 12), car occupants are 31% (n = 9), pedestrians 17% (n = 5) and cyclists 10% (n = 3). Thirty-three percent of PTW occupants (n = 4) are between 26 and 30 years, 25% (n = 3) are between 16 and 20 years. Seventy-five percent (n = 6) of the car occupants are drivers with a mean age of 40.5 years (S.D. 15.8).

### Accident and vehicle configurations

The most frequent road users involved in serious accidents are car passengers 49% (n = 25) followed by PTW users 25% (n = 13), pedestrians 10% (n = 5), cyclists 8% (n = 4), van passengers 6% (n = 3) and buses 2% (n = 1).

The main road accident configurations that have produced a serious injury are “car to PTW” crashes 25% (n = 7), “pedestrian run over” 17,9% (n = 5), “car-to-car” 17.9% (n = 5), “single vehicle PTW” 10.7% (n = 3), “single vehicle car” 7.1% (n = 2), “car-to-bicycle” 7.1% (n = 2), “van-to-PTW” 7.1% (n = 2), “car-to-van” 3.6% (n = 1), PTW-to-bicycle” 3.6% (n = 1).

In the “pedestrian run over” crashes, the vehicles most frequently involved are car 60% (n = 3). Within the PTWs (n = 12) the majority are motorcycles (67%) and the remaining are mopeds (33%).

The main vehicle-to-vehicle collision configurations are the “head-on” and “head-on side” crash 45% (n = 10), followed by “side” and “nose-to-tail” crashes 5% (n = 1). While in the “car to PTW” configuration, 57% of crashes are head-on collision.

### Injury types and severity

In the twenty-nine major traumas analysed, the ISS ranged from 9 to 38 with a mean value of 24.2 (SD 8.7), and NISS ranged from 12 to 5 with a mean value of 33.6 (SD 10.5). The injured included in this paper spent between 3 and 44 days in the hospital (mean 10.6 days, SD 7.9) and between 1 and 34 days in the intensive care unit (mean 14, SD 13.66).

**Figure 16:**
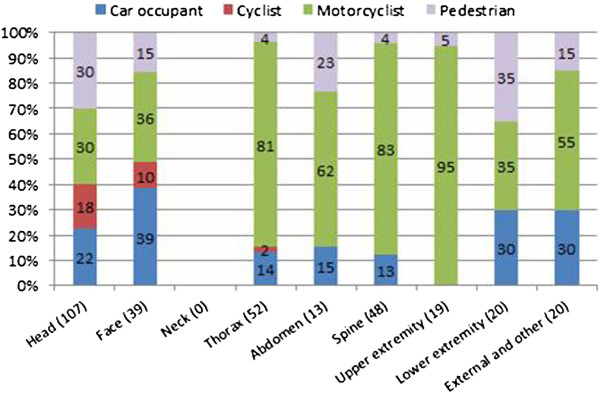
Percentage of injuries by body according to the type of rod user.

Figure 16 shows the percentage of injuries by body part according to the type of road user. Injuries to the head and to the face are prevalent in all users, while neck injuries are absent in the entire sample.

**Figure 17:**
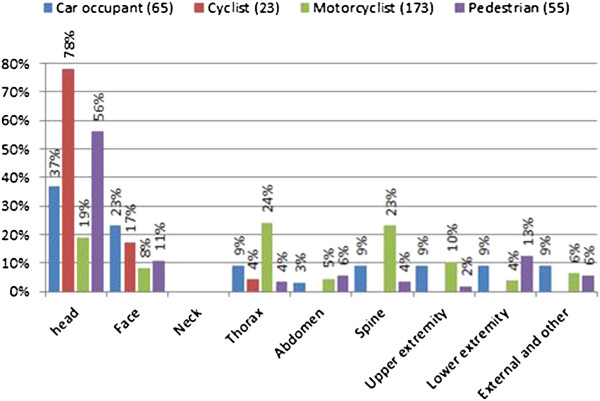
Road user injuries distribution by body part.

Riders-and-pillion-passengers and pedestrians are the road users that reported injuries in all body regions. In the former, the most frequent injuries are to the thorax (24.3%) followed by the spine (23.1%), the head (19.1%), and the upper extremities (10.4%). In the latter, the body regions most frequently injured are the head (56.4%) and the lower extremities (12.7%) (with AIS < 3) (Figure 17).

For cyclists, the body regions most subject to injuries are the head (78.3%), the face (17.4%), and the thorax (4.3%). Finally, the head (36.9%), and the face (23.1%) are the body regions most frequently injured in car occupants, followed by thorax, spine and extremities (9.2%) (Figure 17). Injuries to the upper extremities seem less frequent in cyclists and car occupants than in the other road users.

**Figure 18:**
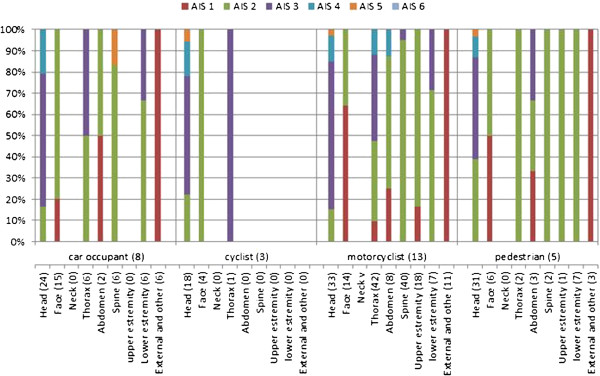
Severity distribution of the injuries by body part according to the type of road user.

Analysing the severity distribution (percentage) of injuries by body part according to the type of road user (Figure 18), the most serious damages have an AIS score equal to five. This level of seriousness is not widespread, but is present in all road users.

PTW riders-and-pillions-passengers, cyclists, and pedestrians have more serious injuries in the head region, while in car occupants the spine is the most severely injured body region. However, in all road users the head is the body part most seriously injured with an AIS3+ in 76.4% (106 lesions). In the thorax, 51% (51 lesions) have an AIS3 + .

The main objects that have produced a high percentage of injuries in the VRUs (Table 3) are asphalt pavement (29.4%), car front bumper (15.7%), car windshield (9.8%), car windshield header rail (8.9%), curb (6%), car A pillar (5.5%) and pole/post (5.1%). The highest values of the AIS (4 and 5) are due to impact with pavement and car windshield header rail.

**Table 3:** Frequency of injuries and severity by object impacted according to the VRU

**Object impacted**		**AIS score**					**Total**
		**1**	**2**	**3**	**4**	**5**	
Asphalt pavement	Count	17	27	17	6	2	69
	%	48,6%	22,7%	27,0%	37,5%	100,0%	29,4%
Barrier, guard rail	Count	3	1	2	0	0	6
	%	8,6%	,8%	3,2%	0,0%	0,0%	2,6%
Front edge or bumper	Count	2	26	7	2	0	37
	%	5,7%	21,8%	11,1%	12,5%	0,0%	15,7%
Curb	Count	3	8	3	0	0	14
	%	8,6%	6,7%	4,8%	0,0%	0,0%	6,0%
External rear view mirror	Count	0	2	0	0	0	2
	%	0,0%	1,7%	0,0%	0,0%	0,0%	0,9%
Front edge or side of bonnet hood)	Count	0	2	1	0	0	3
	%	0,0%	1,7%	1,6%	0,0%	0,0%	1,3%
Fuel tank	Count	1	2	1	0	0	4
	%	2,9%	1,7%	1,6%	0,0%	0,0%	1,7%
Grab rails/ hand holds	Count	0	2	0	0	0	2
	%	0,0%	1,7%	0,0%	0,0%	0,0%	0,9%
Helmet	Count	0	6	2	2	0	10
	%	0,0%	5,0%	3,2%	12,5%	0,0%	4,3%
Pole/ post	Count	3	5	4	0	0	12
	%	8,6%	4,2%	6,3%	0,0%	0,0%	5,1%
Side of bonnet (hood), edge	Count	0	0	1	0	0	1
	%	0,0%	0,0%	1,6%	0,0%	0,0%	0,4%
Top of bonnet, rear	Count	0	4	0	0	0	4
	%	0,0%	3,4%	0,0%	0,0%	0,0%	1,7%
Top of bonnet(hood),front	Count	0	1	3	1	0	5
	%	0,0%	,8%	4,8%	6,3%	0,0%	2,1%
Tree	Count	0	7	2	0	0	9
	%	0,0%	5,9%	3,2%	0,0%	0,0%	3,8%
Upper A-pillar	Count	1	6	5	1	0	13
	%	2,9%	5,0%	7,9%	6,3%	0,0%	5,5%
Windshield header rail	Count	2	7	9	3	0	21
	%	5,7%	5,9%	14,3%	18,8%	0,0%	8,9%
Windshield surface	Count	3	13	6	1	0	23
	%	8,6%	10,9%	9,5%	6,3%	0,0%	9,8%
**Total**	Count	35	119	63	16	2	235
	%	100,0%	100,0%	100,0%	100,0%	100,0%	100,0%

Analysing the source of head injuries in PTW riders-and-pillions-passengers, as seen in Table 4, the highest percentage of injuries was caused by impact against the road surface (38%) and the windshield header rail (31%). Cerebral injuries occurred from all impact sources shown in the table due to the fact that the brain is more sensitive to the inertial forces caused by sudden accelerations and decelerations than the skull base or vault. The highest number of base fracture is due to the impact with windshield head rail of the car opposite.

**Table 4:** PTW occupants: frequency of head injuries and its causes

	***Impact object***					***Total***
	**Asphalt / pavement**	**Barrier /guard rail**	**Curb**	**Pole/post**	**Windshield header rail**	
Base (basilar) fracture	25.0% (2)	0.0% (0)	0.0% (0)	0.0% (0)	75.0% (6)	100% (8 cases)
Cerebrum	36.0% (8)	14.0% (3)	14.0% (3)	18.0% (4)	18.0% (4)	100% (22 cases)
Vault fracture	100.% (2)	0.0% (0)	0.0% (0)	0.0% (0)	0.0% (0)	100% (2 cases)
*Total head injuries*	*38.0% (12)*	*9.0% (3)*	*9.0% (3)*	*13.0% (4)*	*31.0% (10)*	*100% (32 cases)*

Similarly, the main passenger compartment areas more dangerous for car occupants are the front-door–right (26.7%), the windshield (23.3%), the dashboard (13.3%), the steering wheel (8.3%) and the head-rest and passenger (6.7%) (Table 5).

**Table 5:** Frequency of injuries and severity by object impacted according to the car occupants

		**AIS score**					**Total**
		**1**	**2**	**3**	**4**	**5**	
Airbag	Count	2	0	0	0	0	2
	%	18,2%	0,0%	0,0%	0,0%	0,0%	3,3%
Dashboard	Count	0	4	4	0	0	8
	%	0,0%	12,9%	25,0%	0,0%	0,0%	13,3%
Front door - Left	Count	0	1	0	0	0	1
	%	0,0%	3,2%	0,0%	0,0%	0,0%	1,7%
Front door - Right	Count	2	10	4	0	0	16
	%	18,2%	32,3%	25,0%	0,0%	0,0%	26,7%
Handlebars	Count	0	1	0	0	0	1
	%	0,0%	3,2%	0,0%	0,0%	0,0%	1,7%
Head rest	Count	0	0	3	1	0	4
	%	0,0%	0,0%	18,8%	100,0%	0,0%	6,7%
Passenger	Count	0	2	2	0	0	4
	%	0,0%	6,5%	12,5%	0,0%	0,0%	6,7%
Rear view mirror	Count	2	0	0	0	0	2
	%	18,2%	0,0%	0,0%	0,0%	0,0%	3,3%
Roof	Count	2	0	0	0	1	3
	%	18,2%	0,0%	0,0%	0,0%	100,0%	5,0%
Steering wheel	Count	1	3	1	0	0	5
	%	9,1%	9,7%	6,3%	0,0%	0,0%	8,3%
Windshield	Count	2	10	2	0	0	14
	%	18,2%	32,3%	12,5%	0,0%	0,0%	23,3%
**Total**	Count	11	31	16	1	1	60
	%	100,0%	100,0%	100,0%	100,0%	100,0%	100,0%

**Figure 19:**
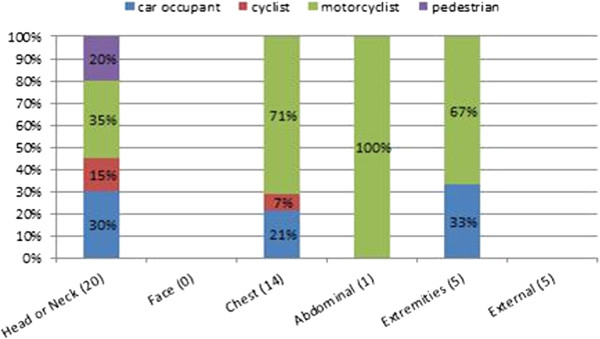
Frequency (%value) of the MAIS3+ for different types of road users.

The frequency percent of the MAIS3+, for different types of road users, on the body region used for the ISS calculation, is shown in Figure 19. It shows how the body regions that report a MAIS3+ are “head or neck”, the chest, the abdominal, and the extremities. The other body parts have a MAIS lower than 3. In each of these body parts, the road user categories with the higher percentages (greater than 30%) are car and PTW occupants, whereas cyclists have a MAIS 3+ only for the head-neck and chest.

## Discussions

The analysis of the state-of-the-art shows that deeper analysis and reconstruction of real-world accidents are an important means for VRUs and automotive safety research.

The correlation of the injuries with their causes and technical parameters allow a better comprehension of injury mechanisms and injury tolerance levels. These studies also give the opportunity to relate the real accident configurations and their consequences to the crash tests results. Structures causing injuries can be recognized at an early stage, and the vehicle’s dynamic response can be identified by the reconstruction. Feedback regarding the road traffic engineering can also be obtained.

In 2011, the successful linkage rate between ICU patients and police information was about 80-85% of the total patients admitted to the ICU for a road accident major trauma. This is mainly due to the retrospective study of the accidents collected and, sometimes, due to the impossibility of knowing which police force has been involved in the road accident detection. In the first case, this working method can lead to insufficiency of significant data for a deeper correlation analysis between dynamics and injuries that lead to exclusion of the case study, The second is due to the fact that several types of police forces in Italy are involved in road accident detection, and many different and unlinked methods are used.

In the urban and metropolitan areas, the range of 16–30 years is the age most subject to serious injury (52%) due to the high percentage of car-to-PTW accident configurations (25%), and given that the PTWs are the vehicles mainly used by this group of people. However, the youngest severely injured are car occupants, with a mean age of 32 years, also if less common.

This can be explained with a more frequent use of dangerous or aggressive behaviour driving/riding compared to elderly people.

Sixty-eight percent of those involved in serious accidents are VRUs. The previous analysis shows that the head is the body region most seriously injured, mainly in pedestrians and cyclists, and the windshield area (centre or upper edge) causes a large percentage (18.7%) of the total injuries incurred. The high incidence of injuries due to ground impact (Table 3) underlines that the second impact is the cause of the greatest number of lesions. This is due to the high quantity of energy that the striking vehicle transmits to the VRU. The five percent of the total injuries sustained by the VRUs are due to the A-pillar impact where, in a total of the 13 lesions, 30% are localized in the head region.

This advises improvement of the vehicle design, e.g. with an wide use of some energy absorbing devices, such as airbags that can be reduce injury risk caused by these structures, without reducing safety performance of the vehicle, by avoiding softening the structures. Alternatively, working on the pre-crash phase with an active system for the collision mitigation based, e.g., on radar and camera acquisition systems. The ground impact suggests the development of new shape of hoods which absorb a greater quantity of energy and release the VRU with a minor speed, so as to reduce the consequences of the second impact with the asphalt.

For the PTW rider-and-pillion, the thorax and the spine are the body regions most frequent injured, while the head is the region with most severe injuries. This latest aspect of the sample analysed is mainly due to the presence of several demi-jet helmets, and of two cases where the helmet became detached after the first impact. This leads to the belief that the use of thoracic protection leads to the reduction of these lesions. Furthermore, the use of full-face helmets reduces the face injury risk, and correct fastening reduces the risk detaching.

The patients spent a mean of 10.6 days in the hospital ward and a mean of 14 days in the ICU. The average daily cost for normal care is calculated at €700, while for intensive care it is €2,000. The average total cost for each patient subject to major trauma (a mean of 24.6 days in the hospital) is equal to €35,400, excluding the cost of physician-staffed ambulance, paramedics or helicopter and ER. Our cost is comparable to what is indicated by Westhoff et al. [52] for Germany (€10,000 - 250,000). Excluding the costs of any period of rehabilitation of the people injured and the intervention of the police forces, the average social expense for the health care of the 29 people was €1,026,600. The social spending is another important facet that it highlights the usefulness of to invest resources for studies and development actions on the mitigation of injuries from road trauma.

Limitations of the present work must be mentioned. Confidence in the results of this study is limited by its low number of road accidents collected so far. Moreover they are heterogeneous in term of accident configurations. Consequently any further and deeper analysis on the data collected is not possible. Further analysis will be conducted as soon as the analysis of all cases acquired during 2011 is concluded. Finally, a multivariate analysis to study the presence of more contributory factors is actually not feasible due to the limited sample size, but the model is still under evolution in this direction.

## Conclusions

A team of ICU physicians, statisticians, and engineers has been setup for the study of real world road accidents in the metropolitan area of Florence.

The information they gather is: environmental, technical and demographic data, treatments, injury score, and follow-up of the person involved in the road accident.

The analysis of the first data collected shows that PTW riders-and-pillions-passengers are subject to high risk of injuries in all body parts, especially on head, thorax and spine. The head is most subject to severe injuries, and the maximum incidence is at cerebral level. This is due to the greater sensitivity of the cerebrum to the inertial force (acceleration) compared to other internal organs. The use of thorax protection and full-face helmets correctly fastened could reduce the severity of the PTW user’s injuries.

The car zones most dangerous for pedestrians and cyclists are the windshield (centre and upper edge), the A-pillar, and the front bumper. The injury severity of pedestrians and cyclist could be reduced by improving the car front design (bumper and hood) and by use of energy absorbers.

## Abbreviations

AD: Anthropometric dummies; AIS: Abbreviated injury scale; CCIS: Co-operative crash injury study; CDC: Collision deformation classification; CIREN: Crash injury research and engineering network; CT: Computer tomography; DMTI: Department of mechanics and industrial technologies; EES: Energy equivalent speed; EMS: Emergency medical services; FDM: Finite difference method; FEHM: Finite-element human model; GCS: Glasgow coma scale; GIDAS: German in depth investigation accident study; EMTRAS: Emergency trauma score; GOS: Glasgow outcome scale; HIC: Head injury criterion; ICU: Intensive care unit; In-SAFE: In-depth study of road accidents in Florence; ISS: Injury severity score; ITARDA: Institute for traffic accident research and data analysis; JARI: Japan automobile research institute; MAIDS: Motorcycle accident in depth study; MBHM: Multi-body human model; NASS: National accident sampling system; NIC: Neck injury criterion; NISS: New injury severity score; OECD: Common international Methodology for in-depth accident investigation; OTS: On the spot; STAIRS: Standardization of accident and injury registration systems; PCC: Passenger compartment classification; PDOF: Principal direction of force; PMHS: Post mortem human subject; PTW: Powered two wheeler; TBI: Traumatic brain injury; TDC: Track deformation classification; TTR: Tuscany trauma registry; VRU: Vulnerable road users; WAD: Wraps around distance

## Competing interests

The authors declare that they have no competing interests (financial or non-financial).

## Authors’ contributions

MP, RS and AP designed the study; SP, DG, MM, RS, MP and AP reviewed the literature; SP; DG and MM collected data; SP and DG performed analysis; SP, DG, MP, GZ and AP wrote draft. All Authors revised and approved the manuscript.

## Pre-publication history

The pre-publication history for this paper can be accessed here:

http://www.biomedcentral.com/1471-227X/13/3/prepub
